# Development and Effectiveness Verification of an Online Career Adaptability Program for Undergraduate Students

**DOI:** 10.3389/fpsyg.2022.857111

**Published:** 2022-03-28

**Authors:** Jihyo Kim

**Affiliations:** Dankook University, Yongin, South Korea

**Keywords:** career adaptability, undergraduates, online, program development, career education

## Abstract

This study developed an online career adaptability improvement program as part of the undergraduate curriculum to improve college students’ career adaptability and verify its effectiveness. This 13-week intervention program, developed using the Korea-Career Adaptability Scale (K-CAS), consists of three domains: (1) knowledge and recognition of the self and work environment, (2) self-directed coping related to career behavior, and (3) environmental interaction for career decisions and adaptation. Two sub-studies were conducted to achieve the research objectives: Study 1 included developing and testing a pilot program and Study 2 quantitatively analyzed the final program to test its effectiveness. In Study 1, preliminary program development and validity were verified, and in Study 2, the effectiveness of the final program was verified. As a strategy to achieve the research purpose, in Study 1, literature review and needs analysis, program design reflecting the results of the needs analysis, validity verification through Delphi survey, preliminary program implementation and evaluation, and the operation of the final program were performed. To test the effectiveness of the program in Study 2, a pre-posttest of career adaptability was conducted on experimental (*n* = 54), comparison (*n* = 56), and control groups (*n* = 53) using the non-equivalent group pretest–posttest design, followed by a program satisfaction survey. This study is meaningful in that it developed an online program to help college students improve career adaptability and identified its effectiveness. This study yielded two results. First, it successfully developed a career adaptability improvement program for undergraduate students, wherein the career development tasks, and the sub-factors of career adaptability were organically interlinked. Second, it verified the statistically significant differences in the career adaptability scores among the experimental, comparison, and control groups. The highest mean score was obtained by participants in the experimental group, followed by the comparison and the control group.

## Introduction

It is no longer possible to create a career plan for the next 30 years within the boundaries of one job or organization (Savickas, 2012). Amidst the ongoing Fourth Industrial Revolution (4IR) and the COVID-19 pandemic, the work world is facing an unpredictable period. The normative idea of lifelong jobs is disappearing, and individuals are faced with many challenges and changes in career choices and paths. This has led to future career-related anxiety and difficulties among undergraduates.

Accordingly, in the field of career education, diverse efforts are being made to provide various career education and support programs for the successful career of college students. Previous studies on career education and group counseling program development for undergraduates have mainly proposed career exploration programs (Kim, 2017; Peng et al., 2020; Lau et al., 2021), career decision-making programs (Kim and Kim, 2016; Lam and Santos, 2018), and career self-efficacy programs (Park and Lee, 2015). Conventional approaches mostly involve connecting individual characteristics and job requirements, with individual selves, based on the trait-factor theory proposed by Parsons (1909). This the mainstream practical career education is still used to help students better understand themselves, explore the work world, and select an occupation matching their personality based on rational inference. However, career education based on the trait-factor theory fails to consider the variability and complexity of the future work world, interactions among numerous environmental and contextual factors that affect career choice and development, and individual traits constantly fluctuating due to changing life circumstances (Kim, 2020). However, since the future world of work is rapidly changing and has uncertainties that are difficult to predict, it would be required for undergraduates to be equipped to appropriately cope with and adapt to changing career paths. In this regard, there is a need for a new program that can improve the ability to cope with unpredictable situations caused by changes in the work environment, not the existing career program that focuses on career decision-making for career selection.

In accordance with this need, studies have developed programs to improve career adaptability among undergraduates, workers, and women affected by career interruptions (Koen et al., 2012; Choi, 2016; Kim, 2016; Eryilmaz and Kara, 2018, 2020; Kim and Jang, 2019). These programs, however, reflect a results-oriented perspective such as job preparation behavior and academic performance, which is distinct from the career support perspective that considers the entire individual’s life. Thus, it is difficult to find a program that focuses on developing the ability of college students to appropriately cope with the problems or changes they face in performing developmental tasks.

In this regard, this study addressed three focus areas not considered in previous studies. First, it developed a program that reflects all key areas required for successful career development in Korean undergraduates. This program consists of three domains of career adaptability based on the developmental tasks of college students: (1) knowledge and recognition of the self and work environment; (2) self-directed coping related to career behavior; and (3) environmental interaction for career decisions and adaptation (Kim J. 2021). Currently, available career adaptability programs include contents related to goal setting, decision-making, information search, and self-efficacy based on constructs such as concern, control, curiosity, and confidence (Koen et al., 2012; Choi, 2016). This study adds the element of social environment interactions, required for undergraduate students to perform career development tasks, which has been overlooked in existing career adaptability programs. It is necessary for adequately performing developmental tasks incumbent on undergraduates, and for predicting adaptive behavior as part of a future workforce. Therefore, it is differentiated in that this program considers the overall adaptive factors required for successful career development task performance, with a focus on difficulties currently encountered by undergraduates in performing these tasks.

Second, since the provided program is an educational program linked with the career adaptability scale for Korean university students, it was designed to involve other treatments appropriate for diagnosis. Its most distinctive feature is that it can provide a learner-centered customized program based on the baseline measurement results (pretest) because it is composed of items reflecting the individual career adaptability scores in the program development stage. In this regard, there is a need to provide customized career consulting by increasing access to individual career information and designing a program tailored to individual needs, breaking away from the conventional uniform format of a special lecture course for many students (Jung et al., 2015). Accordingly, universities should provide various programs as part of the resources available to students, to properly respond to changing career paths and work environments and help them establish their career paths. In this context, an educational program linked to a diagnostic tool is applicable to individually customized career consulting. It enables appropriate educational treatment and counseling suitable for the learner’s diagnosis.

Third, due to the persistent COVID-19 pandemic, a web-based program was developed as an alternative to a conventional classroom program. A literature search for web-based career education programs indicated that most programs were developed as auxiliary tools (e.g., online psychological tests for understanding the self or for web-based career information). Studies on web-based mentoring-type career education programs are mostly for pre-college students and few online career education programs are for college students (Lim and Jang, 2002; Yang et al., 2010; Song and Lee, 2011). In addition, this format is more sustainable than a one-time operation and can be adapted to build a wide range of online career education contents, to achieve a sustainable career education. Therefore, this study aims to develop an online career adaptability program as a way to build various online career education contents to enhance career education.

Overall, this study sought to develop and test the effectiveness of a career education program that can improve undergraduates’ career adaptability. To this end, two research questions were formulated: (1) what is the composition of a program designed to improve undergraduate students’ career adaptability? (2) What are the changes in the career adaptability level of college students who participated in the program?

## Theoretical Background

### Concept of Career Adaptability and Intervention Plan

In the drastically changing future work environment, career development is shifted from the organization to the individual. Therefore, it seems necessary to cope adaptively with the environment and have a flexible attitude.

The career adaptability concept began with career maturity (Super and Knasel, 1981) in the career development theory (Super, 1955) and was substantiated by Savickas (1997). According to the career development theory, the career maturity concept consists of career planning, career exploration, decision-making, work world knowledge, knowledge about preferred occupational groups, and specifically, the extent to which an individual can cope with any given problem commensurate with age (Fouad, 1988). Since the term “maturity” is associated with the growth process (Critchley, 1970), the term “career maturity” was replaced by “career adaptability” due to the inappropriateness of its application among adults (Super and Knasel, 1981). Later, Savickas (1997) specified the career adaptability concept and defined it as a predictable measure, like preparing for a job and performing it, and also as readiness to cope with unpredictable situations triggered by work environment changes.

Studies have conceptualized career adaptability for different subjects. Savickas (2005) established career adaptability central to the Career Construction Theory (CCT). CCT postulates that individuals construct their own lives and career paths by giving meaning to their career-related experiences (Maree, 2013). Savickas (2005) defined career adaptability as a psychosocial construct that denotes an individual’s readiness and resources to cope with anticipated career development tasks, transitions, and personal career-related traumas. In a study with Korean undergraduate students, Jang and Kim (2009) defined career adaptability as the attitude and ability to successfully adapt to various career transitions, and readiness for career decisions, transitions, and adaptation. Considering interactional factors between the work world and Korean students’ career development tasks, Kim J. (2021) defined career adaptability as (1) career development tasks required for a successful career transition, (2) adaptation and self-regulation strategies to appropriately cope with career development tasks and future career tasks necessary for a successful career transition and (3) adaptative and self-regulatory strategies to cope appropriately with future career tasks.

Studies related to career adaptability have been conducted extensively with workers, adults, college students, high school students, out-of-school adolescents, and women with career interruptions (Savickas, 1997; Ployhart and Bliese, 2006; Keum et al., 2008; Creed et al., 2009; Jang and Kim, 2009; Kim J. 2021). Studies on undergraduate students’ career adaptability and related variables reveal career adaptability is associated with social support, career concern, calling, career decision, self-efficacy, and career barriers. These may be regarded as factors with exploratory power for future job satisfaction and successful career transitions (Savickas, 2002, 2005; Creed et al., 2009; Douglass and Duffy, 2015). Therefore, career adaptability is an important concept that influences college students’ future career design. However, very little research has focused on improving career adaptability through career education programs. Studying the practical effect of a career adaptability improvement program is important for the successful development of such a program.

### Career Adaptability Programs

This subsection examines previous studies that designed career adaptability improvement programs for college students and adults and tested their effectiveness. Table 1 outlines the analysis results of the components of career adaptability programs for college students and adults.

**Table 1:** Content areas of career adaptability programs.

Construct	Activity	(1)^a^	(2)^b^	(3)^c^	(4)^d^	(5)^e^	(6)^f^	(7)^g^	(8)^h^
Self-understanding to establish identity	Exploration of interests	1	1		1			1	1
	Exploration of values		1		1		1	1	
	Personality exploration	1	1		1		1	1	1
	Career exploration and self-understanding to establish identity; gaining insights into personality, aptitude, and interest through career process				1			1	
	Exploring the dream history; discovering positive self				1		1		
	Enhancing the career-related interest and intention to implement				1	1			
	Life-cycle experiences: enhancing the self-understanding to establish identity and confidence; sharing career success stories				1		1		
	Writing a want-have list						1		
Work environment and information search	Understanding occupational trends and required abilities	1							
	Finding positive beliefs for the future	1							
	Awareness of the importance of career information and search	1				1	1	1	1
	Inquiring into job requirements; exploring a role or workers	1					1		
	Exploring a career path for the field of interest	1							
	Exploring a lifetime role	1		1	1		1		
Job search skills and interpersonal relationship	Learning how to acquire information on the desired occupation							1	
	Learning interview skills							1	
	Learning business manners							1	
	Coping with conflict situations at work							1	
	Learning the importance of job search skills and expression	1							
Goal setting	Understanding happiness, career, and meaning of life	1							
	Understanding the need for career goal and goal setting	1			1	1		1	
	Enhancing chances by specifying the goal; action planning	1			1	1			1
	Linking the career goal with the learning objective	1							
	Writing a future cover letter to CV; imagining the future						1		1
Decision-making	Exploring career aptitude and knowing the aptitude to develop	1	1						
	Understanding the importance of decision-making and enhancing the rationality of decision-making	1				1			1
	Action goal setting and implementing it; short/long-term planning	1	1	1			1		1
	Focusing on a goal	1		1			1		
Coping	Exploring the career barriers	1		1	1	1			
	Breaking inner walls and setting up coping strategies	1			1	1			
	Knowing the problem-solving ability and applying it		1	1					1
Support	Analyzing the social support system (emotional, evaluative, information support sources)				1		1		1
	Expanding social support sources; career mentoring				1				1

a Choi (2016).

b Eryilmaz and Kara (2018).

c Eryilmaz and Kara (2020).

d Kim (2016).

e Kim W. (2021).

f Kim and Joung (2018).

g Kim and Jang (2019).

h Koen et al. (2012).

Eryilmaz and Kara (2018) developed a psycho-education program to improve the career adaptability of prospective counselors and tested its effectiveness. This 12-session intervention was held once a week for 90 min each, wherein seven activities were performed: exploration, planning, application of coping skills, self-reliance, social support, decision-making skills, and research. Eryilmaz and Kara (2020) investigated the effects of an undergraduate course (guidance and psychological counseling) on freshmen (1st-year) students’ career adaptability. This 12-session program consisted of sub-dimensions including career exploration, curiosity, confidence, career planning, and interest. Koen et al. (2012) developed training for college graduates designed to improve career adaptability resources to enhance chances to find suitable jobs during the school-to-work transition period. The training contained four sections: (1) knowledge of the self, (2) occupational environment knowledge, (3) general implementation of the self-concept into the occupational environment, and (4) concrete implementation of the self-concept into the occupational environment.

In Korea, studies have recently been conducted to improve career adaptability during changes in career development or other problems posed in the context of career support throughout an individual’s life cycle.

Career adaptability programs have also been developed for enhancing career adaptability among various populations, such as women with interrupted careers, mature female students, and college students. These programs’ effectiveness has also been tested. Kim (2016) administered and tested the effectiveness of a career adaptability program for women with an interrupted career. The program included content areas of a job competency enhancement program, structured to help students actively construct their career paths by exploring lifetime themes. Choi (2016) developed and tested the effectiveness of a career adaptability improvement program for college students based on Savickas’ career adaptability theory. This program was composed as an education program aimed at goal setting, decision-making, information search, and self-efficacy in accordance with concern, control, curiosity, and confidence, respectively—the four constructs of career adaptability proposed by Savickas (2005). Kim and Joung (2018) administered a group counseling program for middle-aged female learners to investigate its effect on career adaptability. The content areas of this 10-session program were: rapport formation, understanding of the self, environmental exploration, career interest exploration, career goal setting and activity selection, and program evaluation. Kim and Jang (2019) developed and tested the effectiveness of a customized group career program to improve college students’ career adaptability. Nine sessions were provided, of which five were offered as the basic program (self-understanding to establish identity) and four as the optional program (job search skills and interpersonal relationship). The content areas of the basic program were: career adaptability diagnosis, orientation, exploring interests, exploring aptitudes and values, exploring personality and preferred work environment, and exploring career and incorporation of self-understanding. Kim J. (2021) developed and tested the effectiveness of a peer career counselor training program to improve college students’ career adaptability. In the program, the four subscales of career adaptability (concern, control, curiosity, and confidence) and the four stages of the experiential learning cycle were organically linked.

The present study analyzed the main contents of the career adaptability programs developed previously, to structure the program within the scope of three domains: knowledge and recognition of the self and work environment, self-directed coping related to career behavior, and environmental interaction for career decisions and adaptation.

## Overview of the Studies

**Figure 1:**
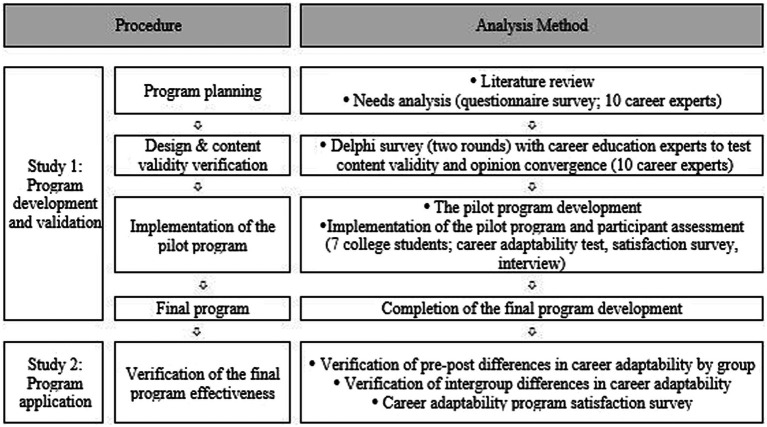
Research procedure and method.

This study aims to develop an online career adaptability improvement program for undergraduate students. Figure 1 illustrates the procedure for program development. Study 1 was conducted to develop a pilot of an online career adaptability improvement program and test its effectiveness. Quantitative analysis was performed in Study 2, to test the effectiveness of the final program.

### Study 1: Development and Validation of the Program

#### Methods

Based on the program development model proposed by Sussman (2001), the career adaptability improvement program for undergraduate students was developed in five steps: (1) literature review and needs analysis, (2) design and developments of a pilot program, (3) content validity verification, (4) pilot program implementation, and (5) final program operation.

##### Participants

###### Needs Analysis

To investigate career adaptability needs, an open-ended questionnaire was administered to 10 career education experts. According to previous studies, the number of expert panels was suggested between 10 and 15 (Saaty, 2000), which is suitable for minimizing errors and ensuring reliability. Therefore, in this study, the panel was selected in consideration of the representativeness, appropriateness, professional knowledge ability, sincerity of participation, and the number of participants.

###### Delphi Survey

A Delphi survey was conducted to seek opinions from experts and test the content validity of the career adaptability improvement program. Two rounds of the Delphi survey were conducted with 10 career education experts who had experience in career education and counseling programs for college students.

###### Pilot Program

Participants (*n* = 7) were undergraduate students studying in a South Korean private university, who voluntarily agreed to participate in the program after reading its content in the career design and self-development curriculum and who had previously participated in a career program. For the pilot program, participants were recruited online, and about 6–8 applicants for the program were recruited (Loo, 2013), and a total of 7 were participated, which is considered an appropriate number of people to share feedback on programs for each session. Reward was paid to program participants. Detailed explanations of the study were provided and informed consent was obtained from all participants. After completing the pilot program, they also participated in a satisfaction survey and in-depth interviews regarding the program contents.

##### Procedure

###### Literature Review and Needs Analysis

This study was approved by the IRB of the affiliated institution. Based on a literature review on career constructivism, career adaptability, career adaptability programs, and career education programs for college students, ideas were collected in a program development meeting. Furthermore, a survey was conducted to determine the need for the program and the content and method with an expert and a student group for improving undergraduates’ career adaptability. The student group survey consisted of five items related to career adaptability, career-related concerns, career areas that require support, career support experience in college, and special requests related to the career program. The expert group survey consisted of five items related to career competencies required to improve career adaptability, the need for a career adaptability program, contents to be considered when developing a career adaptability program, career adaptability program operation mode, and suggestions for effective program operation. Responses were collected using an open-ended questionnaire, sent by e-mail to all participants. The content was analyzed based on the responses.

###### Design and Development of the Pilot Program and Its Content Validity Verification Through a Delphi Survey

The program was designed based on the analysis results of previous studies (Koen et al., 2012; Choi, 2016; Kim, 2016, Kim W. 2021; Eryilmaz and Kara, 2018, 2020; Kim and Joung, 2018; Kim and Jang, 2019). In the absence of prior studies related to this study, the Delphi technique was applied to the study based on the judgment that it is most efficient and reasonable to develop the program through the opinions of experts in related fields, since the Delphi method was conceived as a group technique whose aim was to obtain the most reliable consensus of opinion of a group of experts by means of a series of intensive questionnaires with controlled opinion feedback. Delphi surveys are usually conducted in 3rd to 5th rounds until panel consensus is reached (Khorramshahgol and Moustakis, 1988; Lee, 2016), but the Delphi survey in this study was conducted twice in total since it was judged that consensus was reached among the panelists in the second round based on the statistical index.

###### Implementation Pilot and the Development of the Final Program

Opinions on program participation were collected from students who voluntarily participated in the pilot program, and their post-participation changes were examined. The pilot program was administered intensively: eight sessions in total, three sessions a day lasting 60 min each. Each session was administered online, in real-time, using Google Drive and Zoom. A satisfaction survey was conducted after each session. A pre-posttest was conducted on the participants’ career adaptability, and a satisfaction survey on the program was conducted after the program ended. In the last session, program participants shared their experiences and opinions regarding the program. Through the above procedures, the final program was developed.

##### Instruments

###### Career Adaptability Test

Career adaptability was measured using the K-CAS developed by Kim J. (2021). The test tool comprised 44 items across three domains (knowledge and recognition of the self and work environment, self-directed coping related to career behavior, and environmental interaction for career decisions and adaptation) and nine sub-factors (self-understanding to establish identity, search for a work environment, positive career beliefs, coping with career-choice crisis, career preparation behavior, ability to coordinate career goals, ability to cope with environmental stress, social responsibility, and ability to utilize social resources) is designed to measure career adaptability among South Korean undergraduate students. Each item is rated on a four-point scale; higher scores indicate higher levels of career adaptability. The internal consistency of each sub-factor in this study was: Cronbach’s α for self-understanding to establish identity = 0.774, search for a work environment = 0.723, positive career beliefs = 0.833, coping with career-choice crisis = 0.839, career preparation behavior = 0.788, ability to coordinate career goals = 0.739, ability to cope with environmental stress = 0.778, social responsibility = 0.893, and ability to utilize social resources = 0.828.

###### Program Satisfaction Survey

For the program satisfaction survey, the Client Satisfaction Questionnaire (CSQ) developed by Larsen et al. (1979) was used after modifying some items. Students who participated in the pilot program completed the program satisfaction questionnaire survey. Responses to individual items (appropriateness of the program content, adequacy of the activities, appropriateness of program operation, and amount of help received) were rated on a five-point Likert scale (1 = *strongly disagree*; 5 = *strongly agree*). Participants also responded to an open-ended question about their opinions and experience of the study.

###### Delphi Survey

Two rounds of expert-panel Delphi surveys were conducted to test the content validity of the program. In Round 1, expert opinions on the validity and need for revision were collected regarding the (1) definition and basic direction of the program, (2) goal and objectives of the program, (3) association between career adaptability sub-factors and positive psychological factors, and (4) educational content areas and elements. In Round 2, expert opinions for revision were collected regarding the (1) purpose and goals of the revised program, (2) content composition of the entire program, (3) program operation method, (4) contents organization of each program, and (5) program appropriateness for each session. Initially, a three-round Delphi survey was planned for content validity verification, however, Round 2 yielded a sufficiently high content validity score and degree of consensus among the experts. Considering the content validity verification results, a 13-session program was finally constructed with three career adaptability domains (knowledge and recognition of the self and work environment, self-directed coping related to career behavior, and environmental interaction for career decisions and adaptation) and nine career adaptability sub-factors (self-understanding to establish identity, search for a work environment, positive career beliefs, coping with career-choice crisis, career preparation behavior, ability to coordinate career goals, ability to cope with environmental stress, social responsibility, and utilizing social resources). Participants were recruited for the final program. The program was administered from September to November 2021, followed by effectiveness testing.

##### Analysis

The final program was developed following the literature review of career adaptability improvement programs and needs analysis, design and development of the pilot program, the content validity verification of the pilot program through a Delphi survey, and the implementation of the pilot program. The needs analysis was conducted using an online open-ended questionnaire survey. The survey was conducted online, and the open-ended questionnaire responses were categorized based on the theme, and the analysis was conducted in an iterative and inductive way by adding categories to new themes that did not belong to the categories and continuously comparing them with the previous ones (Merriam, 2009).

The expert-panel Delphi method tested the content validity of the career adaptability improvement program. The two-round expert panel survey data were analyzed by calculating the mean (*M*) and *SD* of each item. Item content validity ratio (CVR) was calculated using the internal validity calculation formula developed by Lawshe (1975). CVR indicates the proportion of participants considered appropriate by raters regarding a specific item. The minimum CVR value depends on the number of Delphi panels, and validity can be recognized only when an item has a CVR value greater than or equal to the number of Delphi panels at the significance level of 0.5. Since there were 10 Delphi panelists in this study and the minimum CVR value was 0.62, items with a CVR value lower than this reference value were revised because they did not meet the criterion for content validity.

For effectiveness testing of the pilot program, repeated measures ANOVA (RM ANOVA) was used. The pre-post-differences in career adaptability scores were examined using a Chi-square (*χ*^2^) statistic, and program satisfaction was measured by analyzing the response rate.

#### Results

##### Needs Analysis

A needs analysis for a career adaptability program was conducted with the 10 expert panelists. The interview results derived five semantic categories. First, career competencies required to improve career adaptability include the “psychological process of successfully coping with inevitable changes and unpredictable needs,” “coping ability in the face of a changing environment,” “self-regulatory strategies to adapt to the work world,” “ability to acquire and utilize knowledge and to learn and utilize cutting-edge technology,” and “ability to obtain and utilize information from social capital.” Second, regarding the need for a career adaptability program, 70.0% of panelists chose “*strongly agree*” and 30.0% chose “*agree*,” indicating that all panelists recognized the need for the program. Third, regarding factors to be reflected in the development of a career adaptability program, panelists recommended “top-down program composition (career vision → understanding of the self → information search → career goals → implementation plan),” “addition of interpersonal skills or social capital building ability as a program,” and activities that can build “self-determination ability to adapt to changing environment,” “optimism in an uncertain future,” “resilience to rechallenge after failure,” “ability to adjust goals according to circumstances,” and “ability to endure ambiguity.” Fourth, for program operation modalities, panelists found it necessary to “improve communication skills based on team-based activities,” “carry out each session with more emphasis on activities than on theories,” “encourage students to share experiences and provide feedback to one another,” “present activity outcomes in collaboration with team members,” “conduct project-based team classes,” and “check the appropriateness of the online environment and activities.” Fifth, the panelists recommended the following to improve program efficacy: contents encompassing failure, frustration, wandering, abandonment, and overcoming challenges; sharing the experiences of failure and overcoming challenges in different professions; programs to build strength to endure an uncertain environment; and development of a career adaptability program differentiating itself from conventional ones.

Some student responses supporting these ideas are as follows:

“It seems necessary to build the ability to obtain and utilize information through interpersonal elements or relationships to achieve career adaptability improvement. For example, it would be a valuable addition to include activities such as finding mentors and role models for career development and forming supportive relationships or finding someone with access to resources necessary for [a] career choice and requesting help.” (Panelist 1)

“When constructing the entire program, I think it would be good to organize it in a top-down manner. For example, when students are preparing for [a] career choice or goal achievement, they start with a great vision for life and have the ability to set career goals and make career plans through understanding their self and information search, [and subsequently] make implementation plans (along with barriers). I think it [is] desirable to organize the program with these sub-factors.” (Panelist 2)

Based on the results of the needs analysis, a pilot program was developed. Then for the content validity verification of the program, a Delphi survey was conducted with career education experts.

##### Delphi Survey

Table 2 presents the results of the two rounds of the Delphi survey about the 24 items related to program design such as the program’s purpose, its goal, linkage between the sub-factors of career adaptability and positive psychological factors, program content areas and elements, and program operation modalities. The mean value for the program in Round 1 of the Delphi survey was 4.40 (*SD* = 0.89), slightly higher than the target value of 4.00–5.00 (*SD* = 0.00–1.23). The mean of the linkage between the sub-factors of career adaptability and positive psychological factors was 4.80 (*SD* = 0.42) and that of the program content areas and elements was slightly high, at 4.20–4.80 (*SD* = 0.45–0.89). The CVR ranged from 0.40 to 0.80. Those falling short of the threshold value of 0.64 calculated by applying the minimum CVR for 10 panelists, as proposed by Lawshe (1975), were judged to have insufficient content validity. Accordingly, elements of “the goal of positive career beliefs,” “program content areas and elements,” “self-understanding to establish identity: strengths, curiosity, values, interests, understanding of the work environment, social responsibility, and coping with career-choice crisis,” and “appropriateness of program operation” were found to need revision. Based on panel opinions on each item, the program goal was revised by converging opinions that state to revise the program goal to properly link career adaptability sub-factors and the goal of the program. Additionally, opinions such as “a clear definition of positive career beliefs” and “redefinition of job environment exploration including analysis of job environment and trends” were presented regarding the program objectives. Regarding program content areas and elements, there was an opinion expressing a need to add practical applications of “the effect of work on life” and “thinking about work from a critical point of view.” It was also pointed out that the program requires distinctive features taken from career education based on Parsons’ theory and Seligman’s positive psychology, which were adopted by partially revising the part in question. Consequently, activities such as “collage expression of happiness,” “treasure hunting to find a gem that makes me happy,” “thinking about the work-life relationship,” “reading future issue analysis reports and understanding the work world in social changes,” and “looking for my role as a member of society” were included. Moreover, Round 1 of the Delphi survey on the appropriateness of the online program operation mode, indicated the CVR at 0.60, revealing low content validity. This problem was solved by revising some activities to make them suitable for an online program operation by adopting opinions of the panelists that some of the contents appeared to be difficult to apply to online classes. As a result of the responses to the revised, second round Delphi questionnaire, the mean score of the linkage between the goal, objectives, career adaptability, and positive psychology sub-factors of the program stood at 5.00 (*SD* = 0.00), that of the program content areas and elements at 4.60–4.80 (*SD* = 0.42–0.52), and that of the appropriateness of the program operation mode at 4.8 (*SD* = 0.42). With 0.80, CVR was high, thus establishing the content validity of the program. Other opinions included the amount of program content, time distribution, and proposals for optional activities considering years and majors.

**Table 2:** Results of Delphi survey for content validation.

Domain	Sub-factor	Results of Delphi round 1			Results of Delphi round 2		
		*M*	*SD*	CVR	*M*	*SD*	CVR
Program goal		4.40	0.89	0.80	5.00	0.00	0.80
Objectives	Self-understanding to establish identity	4.60	0.55	0.80	5.00	0.00	0.80
	Search for a work environment	4.60	0.55	0.80	5.00	0.00	0.80
	Positive career beliefs	4.00	1.23	0.60	4.80	0.42	0.80
	Coping with career-choice crisis	4.60	0.55	0.80	5.00	0.00	0.80
	Career preparation behavior	5.00	0.00	0.80	5.00	0.00	0.80
	Ability to coordinate career goals	4.80	0.45	0.80	5.00	0.00	0.80
	Ability to cope with environmental stress	4.60	0.55	0.80	5.00	0.00	0.80
	Social responsibility	4.80	0.45	0.80	5.00	0.00	0.80
	Utilizing social resources	4.80	0.45	0.80	5.00	0.00	0.80
Content areas and elements of the career adaptability improvement program	Orientation	4.80	0.45	0.80	4.80	0.42	0.80
	Positive career beliefs	4.60	0.55	0.80	4.80	0.42	0.80
	Self-understanding to establish identity: emotions	4.80	0.45	0.60	4.60	0.52	0.80
	Self-understanding to establish identity: curiosity, values, interests	4.20	0.45	0.60	4.80	0.42	0.80
	Search for a work environment	4.40	0.89	0.40	4.80	0.42	0.80
	Social responsibility	4.40	0.89	0.40	4.80	0.42	0.80
	Utilizing social resources	4.80	0.45	0.80	4.80	0.42	0.80
	Coping with career-choice crisis	4.20	0.84	0.40	4.60	0.52	0.80
	Ability to coordinate career goals	4.80	0.45	0.80	4.80	0.42	0.80
	Career preparation behavior	4.80	0.45	0.80	4.60	0.52	0.80
	Integration	4.80	0.45	0.80	4.60	0.52	0.80
Appropriateness of program operation		4.20	0.45	0.60	4.80	0.42	0.80

##### Implementation of the Pilot Program

Differences in the career adaptability pretest and posttest scores were examined for pilot program participants. Of the sub-factors of career adaptability, positive career beliefs (*F* = 10.248, *p* = 0.007), coping with career-choice crisis (*F* = 8.966, *p* = 0.010), ability to cope with environmental stress (*F* = 4.09, *p* = 0.044), and overall career adaptability (*F* = 8.752, *p* = 0.011) revealed statistically significant differences. In the post-intervention program satisfaction survey, “program quality and satisfaction” and “support in problem-solving” resulted in generally high satisfaction with “*neutral*” (0%–26.67%) and “*agree*” (20%–86.67%). The re-participation intention was high with the yes-to-no ratio of 93.3%–6.7%.

##### Completion of the Final Program

As shown in Table 3, the final program included 13 sessions, reflecting the results of content validity testing of the pilot program. The program was finalized after setting the goal for each factor based on the subscales and items of the K-CAS (Kim J. 2021) and previous studies on career adaptability programs (Koen et al., 2012; Choi, 2016; Kim, 2016; Eryilmaz and Kara, 2018, 2020; Kim and Joung, 2018; Kim and Jang, 2019; Kim W. 2021). The program’s purpose is to improve college students’ adaptive ability and self-regulation strategies to respond appropriately to career development tasks. First, by discovering positive emotions and strengths about themselves; second, by helping them create optimistic expectations for the future; and third, through help self-directed career preparation.

**Table 3:** The final version of the career adaptability improvement program for undergraduates.

Session	Session topic	Session goal	Activity
1	Exploring the program needs and objectives	Orientation Enhancing group cohesion	Explaining relationship building and the career adaptability program Forming teams based on similar traits (career decision level, career interest) Self-introduction using positive words Taking baseline tests (career adaptability, needs for the program)
2	Positive career beliefs	Enhancing confidence through positive emotions	“What is happiness to me?” Expressing happy thoughts in a collage Recalling happy moments of past and present, and understanding desire and dynamics in that context Treasure hunt to “find a gem in my life that makes me happy”
3	Self-understanding to establish identity: meaning of work and values	What is work? Understanding work in terms of values and interests	Thinking about work-life relationships, “how work affects my life” Exploring occupational values through values auction
4	Self-understanding to establish identity: strengths	Self-understanding through positive self-awareness	Understanding and exploring greatest strengths through the *via* Survey of Character Strengths Compiling a list of strengths for self-exploration
5	Understanding of work environment	Future work world Understanding work ability and searching for career alternative	Examining the future vision of one’s major Thinking about social changes and trends in the work world from the standpoint of one’s major after reading “Future Issue Analysis Report.”
6	Social responsibility	Finding my role as a member of society	Looking for professionals working the occupation of interest Thinking about things one can do for people marginalized in society
7	Resource utilization ability	Understanding the social support system for my career	Exploring the human and material networking available for career-related support Analyzing the social support system (networking map drawing)
8	Resource utilization ability	Career mentoring	*Via* interviews with career mentors and experts: Building networking ability and proactiveness Sharing experiences of occupational failure and overcoming them
9	Career choice crisis coping ability	Applying strengths and weaknesses in real-life settings	Applying own or others’ strengths and weaknesses to a situation as it unfolds, through a situation board game
10	Career choice crisis coping ability	Understanding and developing my coping ability through my crisis survival skills	Understanding the meaning of a predicament Recalling adversities encountered in the past Sharing experiences of adverse circumstances: acceptance and overcoming
11	Goal adjustment ability	Foreseeing possible career barriers and preparing coping strategies	Understanding the need for lifelong education Sharing experiences of difficulties *via* the dream history Making a career barrier checklist Preparing coping strategies for future difficulties expected
12	Career preparation behavior	Promoting adaptive behavior to make a career chance	Exploring new education, training, and activities in preparation for changes in job requirements Setting up short- and long-term action plans
13	Integration	Drawing life’s vision and direction to feel my own subjective wellbeing	Writing a life’s vision Making a joint collage based on the strengths of individual members Taking a post-program test (career adaptability test, program satisfaction survey, and interviews)

This program has the following objectives: (1) objective understanding of one’s strengths, interests, and values to establish a clear career-related self-concept; (2) job search considering one’s greatest strengths based on the analysis of future work environment and job trends; (3) beliefs in positive results of career choice and goal achievement; (4) coping with career-choice crisis by comparing and analyzing alternatives required for problem-solving and making a comprehensive judgement; (5) planning to participate in new education, training, and various activities in preparation for job requirement changes; (6) adjusting or resetting challenging goals to achieve them in a planned way or need for readjustment in the process of pursuing career goals; (7) exploring coping strategies with positive expectations for overcoming stressful situations experienced in the course of career exploration and pioneering; (8) cooperation with others with a sense of responsibility of one’s role, respecting others’ views and adjusting differences of opinion; and (9) using social networks to achieve career goals or to solve career problems.

The final program was administered to voluntary participants across 13 weekly sessions of 2 h each. Accordingly, the program is composed of 13 session topics: (1) exploring the program needs and objectives; (2) positive career beliefs; (3) self-understanding to establish identity: meaning of work and values; (4) self-understanding to establish identity: strengths; (5) understanding of work environment; (6) social responsibility; (7) and (8) resource utilization ability; (9) and (10) career choice crisis coping ability; (11) goal adjustment ability; (12) career preparation behavior; and (13) integration.

Participants were recruited for the final program and the program was administered online, in real-time, from September to December 2022. Its effectiveness was subsequently tested.

### Study 2: Quantitative Analysis for Effectiveness Test of the Final Program

#### Method

##### Participants

As a result of G power verification, the required number of participants was 159 (53 in each group) when the effect size was 0.25 or higher, the two-tailed significance level was 5%, and the power level was 80%. This calculation was based on the effect sizes obtained in a previous career adaptability program study (Koen et al., 2012; Choi, 2016; Kim, 2016; Eryilmaz and Kara, 2018, 2020; Kim and Joung, 2018; Kim and Jang, 2019; Kim W. 2021). As undergraduate students participated in this study, we had enough power at the start to detect possible effects. To test the effectiveness of the career adaptability improvement program for college students, a total of 163 undergraduates of a South Korean private university were recruited and randomly assigned to the experimental group (participating in the program developed in this study; *n* = 54, 33.1%), comparison group (participating in an existing program; *n* = 56, 34.4%), and the control group (no program exposure; *n* = 53, 32.5%). The participant characteristics were 29 students (17.8%) from the College of Public Talent, 44 (27%) from the College of Science and Technology, 34 (20.9%) from the College of Health Sciences, 27 (16.6%) from the College of Life Science and Resource Science, and 8 (4.9%) from the College of Sports Science. The number of students (4.9%) was 21 students (12.9%) from foreign language colleges. Of the participants, 63 (38.7%) were male and 100 (61.3%) were female.

##### Instrument

To test the effectiveness of the final program and examine the program satisfaction, the K-CAS (Kim J. 2021) and the CSQ (Larsen et al., 1979) presented in Study 1 were used. Additionally, participant perceptions and satisfaction in the experimental group were examined using an open-ended questionnaire. The questionnaire included items related to individual career paths, benefits gained from program participation, program satisfaction, and suggestions.

##### Procedure and Analysis

**Figure 2:**
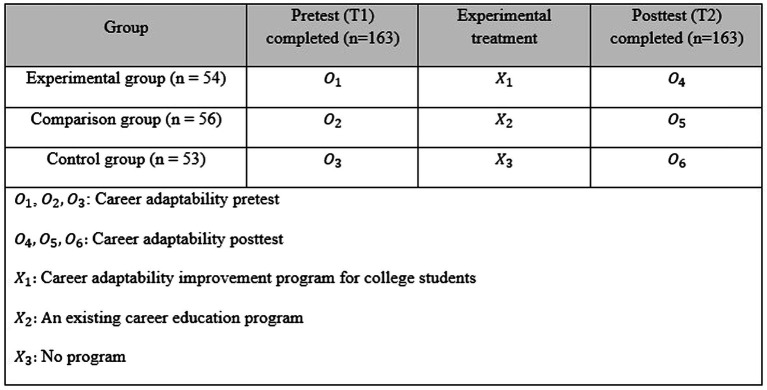
Experimental design for program effectiveness testing.

The non-equivalent group pretest–posttest design was used to investigate the effect of the career adaptability improvement program developed in this study (Figure 2). Both pretest and posttest were administered to all three groups (experimental, comparison, and control). While the experimental group participated in the career adaptability improvement program and the comparison group participated in the career education program designed to improve career maturity through the process of understanding of the self → career search → career goal setting → career preparation, the control group did not participate in any program. The career maturity improvement program was configured by linking individual characteristics and occupational factors as proposed by Parsons (1909) trait-factor theory. That is, it is designed to help students better understand themselves and the work world, and rationally infer and choose a job that best suits them. The program is structured to be administered in 13 sessions composed of activities directly associated with college life. For example, understanding the self by better understanding one’s personality, interests, and needs through MBTI and Holland tests, exploring the work world based on the proper understanding of the specialized fields and jobs, establishing career vision, career development, and life-span design.

Quantitative data were analyzed using the software SPSS Statics 25.0 to examine the program’s effectiveness. Mixed ANOVA was performed to examine career adaptability pretest–posttest timing and the main and interaction effects of the experimental, comparison, and control groups to determine whether changes in participants’ career adaptability scores were statistically significant. Moreover, the effect of the career adaptability improvement program on the learner’s career adaptability was tested using covariance analysis. Baseline scores (pretest) for career adaptability were set as the covariant and post-intervention scores (posttest) of each group as the dependent variable. In addition, a Bonferroni *post hoc* test was performed.

#### Results

##### Normality, Homogeneity, and Sphericity Test Results of the Experimental, Comparison, and Control Groups

The descriptive statistics (mean and SD, skewness, and kurtosis) of the experimental, comparison, and control groups are outlined in Table 4. The results of the Kolmogorov–Smirnov normality test to verify the normality of the experimental group, comparison group, and control group indicated that a normal distribution in the crisis response and goal adjustment were not satisfied in the comparison and control groups. However, the values of skewness and kurtosis were between −2 and 2 (Snedecor and Cochran, 1980). Thus, all three groups were performed assuming a normal distribution.

**Table 4:** Descriptive statistics by group.

Sub-factor	Experimental group			Comparison group			Control group			Levene statistic	*p*
	M(SD)	S	K	M(SD)	S	K	M(SD)	S	K		
Self-understanding to establish identity	2.95 (0.37)	0.95	0.13	2.6 (0.48)	−0.65	−0.44	2.92 (0.41)	−0.32	0.51	1.449	0.238
Search for a work environment	3.09 (0.36)	1.56	1.48	2.66 (0.42)	−0.76	0.09	2.88 (0.37)	−0.79	1.90	1.070	0.346
Positive career beliefs	2.97 (0.43)	0.29	−0.18	2.89 (0.45)	−0.56	−0.12	2.91 (0.36)	−1.00	0.99	1.549	0.216
Coping with career-choice crisis	2.9 (0.38)	1.06	1.78	2.83 (0.27)	−1.50	1.61	2.85 (0.25)	−1.15	1.59	2.140	0.054
Career preparation behavior	2.75 (0.32)	−0.28	0.20	2.65 (0.39)	−0.95	0.67	2.8 (0.4)	−0.06	0.30	1.143	0.322
Ability to coordinate career goals	3.02 (0.39)	1.22	1.36	2.69 (0.41)	−1.44	1.05	2.9 (0.35)	−0.79	1.28	0.136	0.873
Ability to cope with environmental stress	2.69 (0.37)	−0.06	−0.20	2.54 (0.43)	−0.75	0.06	2.66 (0.43)	0.24	0.55	1.433	0.242
Social responsibility	3.34 (0.46)	0.39	−1.66	3.19 (0.55)	−0.47	−0.17	3.29 (0.42)	0.21	−0.62	1.998	0.139
Utilizing social resources	2.35 (0.64)	0.38	−0.85	2.44 (0.67)	−0.17	0.37	2.47 (0.55)	0.07	1.28	1.091	0.339
Overall career adaptability	2.9 (0.23)	0.00	−1.26	2.74 (0.28)	−1.47	1.76	2.86 (0.26)	−0.81	1.87	0.117	0.890

S, skewness and K, kurtosis.

Levene’s statistics (pretest score: Levene value = 0.117, *df* = 2, 145, *p* = 0.888; posttest score: Levene value = 0.511, *df* = 2, 145, *p* = 0.601) were used for the homogeneity test. The test results showed no statistically significant differences in the mean scores between pretest and posttest of each group, which confirmed intergroup homogeneity. Box’s test for homogeneity of covariance matrices revealed that covariance matrices were equal across the three groups, thus, satisfying the equal variances assumption (*M* = 5.034, *F* = 0.822, *p* > 0.05). In the sphericity test, the sphericity assumption was found to be violated (*W* = 0.60, $x^{2}$ (3) = 10.74, *p* < 0.05), and Greenhouse–Geisser ∈ was applied.

##### Pretest–Posttest Comparative Analysis of the Program

The mean and SD of the pretest and posttest scores of the experimental, comparison, and control groups are presented in Table 4 to examine the effect of the career adaptability program on career adaptability level. There were differences in the mean value between the pretest and posttest scores by group with respect to the overall career adaptability score. The experimental group had a higher mean score in the posttest (*M* = 3.11, *SD* = 0.32) compared with the pretest (*M* = 2.9, *SD* = 0.23). The comparison group had relatively high scores in both pretest (*M* = 2.74, *SD* = 0.28) and posttest (*M* = 2.87, *SD* = 0.29). The control group showed little difference (*M* = 2.86, *SD* = 0.26 vs. *M* = 2.80, *SD* = 0.30).

The analysis of the results from the career adaptability pretest–posttest timing and the main and interaction effects of the experimental, comparison, and control groups are outlined in Table 4. The results of examining time-dependent changes in the mean score of career adaptability by measuring the difference between pretest and posttest scores are as follows: with *F* = 12.386 (*p* < 0.001, $\eta^{2}$ = 0.079), changes in the mean career adaptability score over time were found to be significant. Broken down into sub-factors, those that showed significant time-dependent changes in the mean career adaptability score were self-understanding to establish identity (*F* = 21.259, *p* < 0.001, $\eta^{2}$ = 0.135), searching for a work environment (*F* = 13.896, *p* < 0.001, $\eta^{2}$ = 0.093), ability to coordinate career goals (*F* = 5.863, *p* < 0.05, $\eta^{2}$ = 0.041), and ability to cope with environmental stress (*F* = 8.111, *p* < 0.01, $\eta^{2}$ = 0.056).

Regarding the intergroup differences (experimental, comparison, and control groups) in the mean score of career adaptability, with the *F* value of 11.280 (*p* < 0.001, $\eta^{2}$ = 0.135), it was confirmed that there were significant differences. Sub-factors that showed significant intergroup changes were self-understanding to establish identity (*F* = 4.385, *p* < 0.05, $\eta^{2}$ = 0.061), searching for a work environment (*F* = 5.130, *p* < 0.01, $\eta^{2}$ = 0.070), positive career beliefs (*F* = 3.563, *p* < 0.05, $\eta^{2}$ = 0.049), coping with career-choice crisis (*F* = 16.523, *p* < 0.001, $\eta^{2}$ = 0.195), ability to coordinate career goals (*F* = 17.068, *p* < 0.001, $\eta^{2}$ = 0.201), and ability to cope with environmental stress (*F* = 8.961, *p* < 0.001, $\eta^{2}$ = 0.116).

With the *F* value of 7.293 (*p* < 0.01, $\eta^{2}$ = 0.091) for the interaction effect between time and group on the mean career adaptability score, the interaction effect was statistically significant. Sub-factors that showed significant changes in the interaction effect between time and group were self-understanding to establish identity (*F* = 14.020, *p* < 0.001, $\eta^{2}$ = 0.171), searching for a work environment (*F* = 12.679, *p* < 0.001, $\eta^{2}$ = 0.157), coping with career-choice crisis (*F* = 9.635, *p* < 0.001, $\eta^{2}$ = 0.124), ability to cope with environmental stress (*F* = 5.358, *p* < 0.01, $\eta^{2}$ = 0.073), social responsibility (*F* = 5.168, *p* < 0.01, $\eta^{2}$ = 0.070), and utilizing social resources (*F* = 6.738, *p* < 0.01, $\eta^{2}$ = 0.090).

##### Difference Analysis of Group-Specific Career Adaptability

The mean and SD of the career adaptability scores obtained by experimental, comparison, and control groups in the pretest and posttest conducted to examine the effects of the career adaptability program on career adaptability are presented in Table 5. A comparison of the overall career adaptability pretest and posttest scores between the experimental and comparison groups revealed that the posttest score was higher than the pretest score in both the experimental group (*M* = 2.9 → 3.11) and the comparison group (*M* = 2.74 → 2.87). In contrast, the control group showed little difference (2.86 and 2.80). Regarding the changes in the mean pretest and posttest scores for individual sub-factors of career adaptability, an increase in posttest scores was observed in most sub-factors in the case of the experimental group: self-understanding to establish identity, search for a work environment, positive career beliefs, ability to coordinate career goals, ability to cope with environmental stress socially, and responsible behavior in the case of the comparison group. For the control group, minor changes were observed or even lower in some of the sub-factors.

**Table 5:** Pre-post-comparison of the program (RM ANOVA).

Independent variable	Dependent variable	SS	df	MS	*F*	*p*	$\eta^{2}$
Measurement point	Self-understanding to establish identity	2.966	1.000	2.966	21.259	0.000	0.135
	Search for a work environment	2.594	1.000	2.594	13.896	0.000	0.093
	Positive career beliefs	0.355	1.000	0.355	2.358	0.127	0.017
	Coping with career-choice crisis	0.339	1.000	0.339	2.044	0.155	0.015
	Career preparation behavior	0.000	1.000	0.000	0.003	0.959	0.000
	Ability to coordinate career goals	0.615	1.000	0.615	5.863	0.017	0.041
	Ability to cope with environmental stress	1.607	1.000	1.607	8.111	0.005	0.056
	Social responsibility	0.405	1.000	0.405	2.910	0.090	0.021
	Utilizing social resources	0.948	1.000	0.948	3.210	0.075	0.023
	Overall career adaptability	0.730	1.000	0.730	12.386	0.001	0.079
Group	Self-understanding to establish identity	1.021	2.000	0.511	4.385	0.014	0.061
	Search for a work environment	1.262	2.000	0.631	5.130	0.007	0.070
	Positive career beliefs	1.721	2.000	0.861	3.563	0.031	0.049
	Coping with career-choice crisis	5.304	2.000	2.652	16.523	0.000	0.195
	Career preparation behavior	0.906	2.000	0.453	1.686	0.189	0.025
	Ability to coordinate career goals	3.267	2.000	1.634	17.068	0.000	0.201
	Ability to cope with environmental stress	4.387	2.000	2.193	8.961	0.000	0.116
	Social responsibility	0.379	2.000	0.189	1.207	0.302	0.017
	Utilizing social resources	0.819	2.000	0.410	1.647	0.196	0.024
	Overall career adaptability	2.249	2.000	1.124	11.280	0.000	0.135
Measurement point * Group	Self-understanding to establish identity	3.912	2.000	1.956	14.020	0.000	0.171
	Search for a work environment	4.733	2.000	2.367	12.679	0.000	0.157
	Positive career beliefs	0.642	2.000	0.321	2.130	0.123	0.030
	Coping with career-choice crisis	3.201	2.000	1.600	9.635	0.000	0.124
	Career preparation behavior	0.554	2.000	0.277	2.424	0.093	0.036
	Ability to coordinate career goals	0.457	2.000	0.228	2.176	0.117	0.031
	Ability to cope with environmental stress	2.123	2.000	1.061	5.358	0.006	0.073
	Social responsibility	1.439	2.000	0.719	5.168	0.007	0.070
	Utilizing social resources	3.980	2.000	1.990	6.738	0.002	0.090
	Overall career adaptability	0.859	2.000	0.430	7.293	0.001	0.091

SS, sum of squares; df, degrees of freedom; and MS, mean square.

Covariate analysis was performed to determine whether there are significant differences in the mean career adaptability scores between the experimental, comparison, and control groups. The adjusted means of the overall career adaptability scores for the three groups were 3.0, 2.9, and 2.8, respectively. The results of covariate analysis that indicate whether the corrected posttest career adaptability scores were significantly different depending on program participation are shown in Table 6. The statistical significance of the corrected posttest career adaptability scores of the three groups was examined after controlling for the effect of the pretest career adaptability scores. Consequently, participation in the program had a significant positive effect on the corrected posttest career adaptability scores (*F* = 11.963, *p* < 0.001, $\eta^{2}$ = 0.142). The *post hoc* test revealed that the career adaptability of the comparison group was higher than the control group and lower than the experimental group. These results suggested that the career adaptability program has a positive effect on the overall career adaptability among participants in the experimental group. Intergroup comparison of the differences in the career adaptability sub-factors revealed that there were significant differences in all sub-factors except for career preparation behavior. The posttest scores of the experimental and comparison groups were those of the control group in self-understanding to establish identity (*F* = 8.094, *p* < 0.001, $\eta^{2}$ = 0.063), searching for the work environment (*F* = 6.079, *p* < 0.01, $\eta^{2}$ = 0.083), positive career beliefs (*F* = 4.628, *p* < 0.05, $\eta^{2}$ = 0.063), and social responsibility (*F* = 5.249, *p* < 0.001, $\eta^{2}$ = 0.072). In coping with career-choice crisis, (*F* = 17.715, *p* < 0.001, $\eta^{2}$ = 0.208), ability to coordinate career goals (*F* = 9.714, *p* < 0.001, $\eta^{2}$ = 0.126), ability to cope with environmental stress (*F* = 10.162, *p* < 0.001, $\eta^{2}$ = 0.131), and ability to utilize social resources (*F* = 7.645, *p* < 0.01, $\eta^{2}$ = 0.102), the experimental group scored higher than the comparison and control groups.

**Table 6:** Comparison of pre- and post-program scores by group (covariance analysis result).

Construct		T1	T2	TM	SV	SS	df	M2	*F*	*p*	$\eta^{2}$	
Self-understanding to establish identity	G1	2.95 (0.37)	3.15 (0.32)	3.12 (0.06)	Covariance (pre)	1.655	1	1.655	9.073	0.003	0.063	a, b > c
	G2	2.6 (0.48)	3.1 (0.55)	3.16 (0.06)	Group	2.954	2	1.477	8.094	0.000	0.107	
	G3	2.92 (0.41)	2.84 (0.41)	2.82 (0.06)	Error	24.632	135	.182b				
					Sum	29.241	138					
Search for a work environment	G1	3.09 (0.36)	3.12 (0.47)	3.08 (0.08)	Covariance (pre)	0.795	1	0.795	2.858	0.093	0.021	a, b > c
	G2	2.66 (0.42)	3.22 (0.67)	3.26 (0.08)	Group	3.383	2	1.691	6.079	0.003	0.083	
	G3	2.88 (0.37)	2.86 (0.41)	2.87 (0.08)	Error	37.562	135	.278b				
					Sum	41.740	138					
Positive career beliefs	G1	2.97 (0.43)	3.17 (0.39)	3.15 (0.07)	Covariance (pre)	1.633	1	1.633	7.921	0.006	0.054	a, b > c
	G2	2.89 (0.45)	2.96 (0.43)	2.97 (0.06)	Group	1.908	2	0.954	4.628	0.011	0.063	
	G3	2.91 (0.36)	2.87 (0.55)	2.87 (0.07)	Error	28.444	138	.206b				
					Sum	31.984	141					
Coping with career-choice crisis	G1	2.9 (0.38)	3.28 (0.54)	3.28 (0.07)	Covariance (pre)	0.011	1	0.011	0.048	0.826	0.000	a > b, c
	G2	2.83 (0.27)	2.76 (0.44)	2.76 (0.07)	Group	8.339	2	4.170	17.715	0.000	0.208	
	G3	2.85 (0.25)	2.74 (0.47)	2.76 (0.07)	Error	31.775	135	.235b				
					Sum	40.125	138					
Career preparation behavior	G1	2.75 (0.32)	2.83 (0.55)	2.83 (0.07)	Covariance (pre)	5.573	1	5.573	27.487	0.000	0.176	-
	G2	2.65 (0.39)	2.67 (0.51)	2.73 (0.07)	Group	0.765	2	0.383	1.888	0.156	0.028	
	G3	2.8 (0.4)	2.67 (0.43)	2.64 (0.07)	Error	26.156	129	.203b				
					Sum	32.495	132					
Ability to coordinate career goals	G1	3.02 (0.39)	3.21 (0.48)	3.17 (0.06)	Covariance (pre)	1.711	1	1.711	12.552	0.001	0.085	a > b, c
	G2	2.69 (0.41)	2.79 (0.29)	2.84 (0.06)	Group	2.649	2	1.324	9.714	0.000	0.126	
	G3	2.9 (0.35)	2.88 (0.36)	2.88 (0.06)	Error	18.406	135	.136b				
					Sum	22.766	138					
Ability to cope with environmental stress	G1	2.69 (0.37)	3.08 (0.69)	3.07 (0.08)	Covariance (pre)	0.439	1	0.439	1.609	0.207	0.012	a > b, c
	G2	2.54 (0.43)	2.63 (0.45)	2.65 (0.08)	Group	5.546	2	2.773	10.162	0	0.131	
	G3	2.66 (0.43)	2.64 (0.37)	2.64 (0.08)	Error	36.838	135	0.273				
					Sum	42.823	138					
Social responsibility	G1	3.34 (0.46)	3.42 (0.48)	3.39 (0.06)	Covariance (pre)	4.442	1	4.442	23.725	0.000	0.149	a, b > c
	G2	3.19 (0.55)	3.44 (0.5)	3.47 (0.06)	Group	1.965	2	0.983	5.249	0.006	0.072	
	G3	3.29 (0.42)	3.19 (0.43)	3.19 (0.06)	Error	25.461	136	.187b				
					Sum	31.868	139					
Utilizing social resources	G1	2.35 (0.64)	2.79 (0.73)	2.79 (0.73)	Covariance (pre)	3.611	1	3.611	9.381	0.003	0.065	a > b, c
	G2	2.44 (0.67)	2.32 (0.61)	2.32 (0.61)	Group	5.886	2	2.943	7.645	0.001	0.102	
	G3	2.47 (0.55)	2.47 (0.57)	2.48 (0.57)	Error	51.967	135	.385b				
					Sum	61.464	138					
Overall career adaptability	G1	2.9 (0.23)	3.11 (0.32)	3.09 (0.04)	Covariance (pre)	0.883	1	0.883	10.398	0.002	0.067	a > b > c
	G2	2.74 (0.28)	2.87 (0.29)	2.9 (0.04)	Group	2.032	2	1.016	11.963	0.000	0.142	
	G3	2.86 (0.26)	2.8 (0.3)	2.8 (0.04)	Error	12.232	144	.085b				
					Sum	15.148	147					

TM, trimmed mean; SV, sources of variance; SS, sum of squares; and df, degrees of freedom.

##### Program Satisfaction Survey in Experimental and Comparative Groups

Upon program completion, participants in the experimental and comparison groups completed a satisfaction survey regarding program quality and satisfaction, problem-solving, the degree of support in improving career adaptability, and re-participation intention. Participants in both groups responded positively to the quality of and satisfaction with the overall program. However, significant intergroup differences were revealed in the degree of satisfaction with the program need, recommendation intention, support in solving career problems, and support in career adaptability. The ratio of positive answers (“*agree*” and “*strongly agree*”) regarding the degree of satisfaction of the program need was significantly higher in the experimental group (G1) than in the comparison group (G2; G1 = 81.5%, G2 = 51.8%), recommendation intention (G1 = 98.1%, G2 = 83.9%), support in solving career problems (G1 = 87.0%, G2 = 39.3%), and support in career adaptability (G1 = 90.7%, G2 = 58.9%).

To analyze the data of the open-ended questionnaire, thematic analysis procedures were conducted in a non-mathematical and iterative manner such that participants’ responses could be interpreted and described for the reconstruction of data into “a recognizable reality” (Maykut and Morehouse, 1994). Open-ended questions about the perceptions of and satisfaction about program participation resulted in two opinion categories: (1) opinions about individual career paths and (2) opinions about the program. Regarding the effect of the program on an individual career path, participants reported an “increase in positive emotions about the future,” “enhancement of appropriate coping skills for career problems,” “facilitation of career design through the participation experience,” and “discovery of new perspectives for goal setting.” Examples of participants’ responses in this opinion category are as follows:

“In the 4IR era and the COVID-19 pandemic situation, jobs seem to be changing too rapidly, and I was deeply concerned about which job to choose. Now that I have changed my thoughts about the change itself, I feel less anxious, and my confidence has increased.” (Student B)

In the category of opinions and perceptions regarding the program and changes after the participation in the program, participants reported on “the advantage of the program linked with [a] test score,” “the pros and cons of the online career program,” “the advantage of the activities directly related to my own career,” and “better concentration due to activity-centered program operation.” Examples of participants’ responses in this regard are as follows:

“At first, it felt it burdensome to do group activities online, but it was convenient to share career information. [And] It was so nice to be able to communicate with my friends in convenient online classes after missing opportunities to communicate with them.” (Student D)

## Discussion

This study was conducted to develop and test the effectiveness of a career education program to improve undergraduate students’ career adaptability. The major results of this study lead to the following discussion points. First, the results of this study support the findings of previous studies. The career adaptability improvement program developed in this study consisted of three domains pertaining to university students’ career development tasks and nine adaptive factors to properly perform the domain objectives. Moreover, it had a positive effect on career adaptability (Koen et al., 2012; Choi, 2016; Kim, 2016; Eryilmaz and Kara, 2018, 2020; Kim and Joung, 2018; Kim and Jang, 2019). Most existing career adaptability improvement programs (Koen et al., 2012; Choi, 2016; Kim W. 2021) are composed of the four constructs proposed by Savickas: concern, control, curiosity, and confidence. This demonstrates the effectiveness of those programs for various target groups, such as university students (Koen et al., 2012; Choi, 2016; Kepir Savoly, 2017; Kim and Jang, 2019; Kim W. 2021), women with an interrupted career (Kim, 2016), or middle-aged female learners (Kim and Joung, 2018). The results of the present study were consistent with those of previous studies that have developed and tested the effectiveness of career adaptability programs for college students (Koen et al., 2012; Choi, 2016; Kepir Savoly, 2017; Kim and Jang, 2019; Kim W., 2021), wherein participants reported the programs’ positive effects on their career adaptability. However, the program of this study differentiates itself from existing programs as it includes content areas designed to improve the adaptability in performing career development tasks; for example, interaction with the environment and self-directed coping skills related to career behavior.

Second, undergraduate years fall in the late exploring phase along the life-long process of career development. The main developmental tasks in this period are career exploration and decision-making for higher studies or a job search (Super, 1990). As there are individual differences in the career developmental stage for undergraduates, this study focused on increasing the ability to adapt to developmental tasks specific to each individual. A needs analysis with active career experts revealed that Korean university students lack an understanding of the self and need the ability to cope with and overcome difficulties in a self-directed manner. These needs were reflected in the construction of the content areas of the program developed in this study. These considerations show similar approaches to previous studies (Koen et al., 2012; Choi, 2016; Kim W. 2021) that proposed a career adaptability intervention based on the four domains of career adaptability: planning (concern), decision-making (control), exploration (curiosity), and problem-solving (confidence). The proposed program is different from other similar programs as it focuses on college students’ career development tasks from a new approach toward career domains. Moreover, it is constructed with content areas conducive to improving students’ adaptive abilities across various problem areas. In the proposed program in this study, career problems encountered by undergraduate students are divided into three dimensions of career development tasks that individual students can identify as areas in which they need help. This suggested it is possible to design personalized programs according to the students’ career adaptability levels.

Third, the career adaptability improvement program for undergraduate students improved career adaptability. The experimental group, to which the program was administered, showed a significantly higher mean posttest score in career adaptability compared with the control group, which was exposed to no intervention. It also indicated higher career adaptability than the comparison group in some sub-factors. These results supported previous studies which reported that career adaptability programs for college students have a positive effect on their career adaptability (Koen et al., 2012; Choi, 2016; Kepir Savoly, 2017; Kim and Jang, 2019; Kim W. 2021).

Furthermore, differences in pre-post-scores for career adaptability sub-factors were observed between the experimental and comparison groups. This was similar to Kim and Jang (2019) report that indicated intergroup differences in the pre-post-scores for career adaptability vary according to sub-factors. More specifically, they reported that posttest scores of the experimental group were higher than the pretest scores in goal awareness, job performance, and positive attitude. These results provide information on which career adaptability sub-factors were specifically affected by the program. However, there are other studies (Kim W. 2021) that only provide the difference in overall career adaptability by group and measurement point without further details. Therefore, the main focus of this study was on examining which career adaptability factors are improved by the career adaptability improvement program. A comparison of the differences in the posttest scores for the career adaptability sub-factor by group revealed that the experimental and comparison groups scored higher than the control group in self-understanding to establish identity, search for a work environment, positive career beliefs, and social responsibility. In this context, no significant difference was observed between the experimental and comparison groups in the *post hoc* test. In the other sub-factors, however, such as the ability to cope with career-choice crisis, ability to coordinate career goals, ability to cope with environmental stress, and ability to utilize social resources, the experimental group scored significantly higher than the comparison group. These results suggested that the proposed program was effective in improving some career adaptation abilities that could not be improved through existing career education programs. In this respect, the proposed program made a particular contribution to the undergraduates’ unstable and confusing situation in the face of school-to-work transition after graduation by improving their abilities to cope with the career-choice crisis, coordinate career goals, cope with environmental stress, and utilize social resources essential for achieving career development tasks.

### Contribution and Implications

The significance of this study is as follows. First, at this juncture in time when the paradigm for future careers is changing, it developed a career adaptability improvement program that differentiates itself from the existing career education programs. Career education programs that improve coping ability with various career problems and solving career development tasks based on self-understanding are important for lifelong career design that considers the life cycle. Accordingly, this study is expected to contribute to meeting future career education needs. In particular, the proposed program has the advantage of being used immediately in college settings. Although many students need advice or guidance for their career problems, only a small proportion of students visit the on-campus career counseling center for assistance. Therefore, students who participate in this career adaptability improvement program have the opportunity to diagnose their career adaptability and receive a personalized program. This program can be used any time in different faculties related to careers. The career adaptability improvement program for 1st-year can screen students with low career adaptability. Those who need in-depth counseling can be linked with the relevant divisions, such as the student counseling center and the employment and career assistance office on campus.

Additionally, with the increased demand for online career education programs, it can be used as a model for developing on/offline blended career education programs. In this regard, its significance lies in the empirical data accumulated by testing the effectiveness of online career education programs. An online career education program is advantageous as it can overcome physical constraints and educational environments, and can be used anytime, anywhere according to participants’ needs. However, compared to face-to-face lectures, there may be restrictions for some program content. An efficient educational environment and function may not be provided. This aspect requires further discussion.

Second, the program was developed in tandem with the adaptive factors required to properly cope with domains related to college students’ career development tasks in order to improve their career adaptability. This program was composed of educational content corresponding to the overall sub-factors of career adaptability for 1st-year students. This approach is significant as it proposes a program in a practically applicable form to improve students’ career adaptability in academic career courses. Additionally, this approach improved undergraduate students’ career adaptability in various career domains centering on career development tasks that led directly lead to a student-centered intervention. This was because the program can effectively improve the adaptability needed to address student career problems.

Third, this study emphasized developing the program in compliance with a systematic procedure. In the program development stage, the results of an expert panel’s career needs analysis were reflected on, and a Delphi survey was conducted to test the content validity. The results of the pilot program implementation were integrated into the program revision. Therefore, the program was developed by reflecting the opinions of an expert panel currently active in college settings and the needs of actual program participants to enhance the onsite effectiveness.

Fourth, the program effectiveness was tested through quantitative analysis based on experimental design, and through a program perception and satisfaction survey with participants. This collected opinions as feedback for further program revision and improvement. This made it possible to verify the positive aspects of the program related to the career adaptability test, such as the improvement of cooperating ability and the convenience of information sharing through the online career education program administered in the contact-free class setting. However, considering that web-based teamwork may be burdensome in a contact-free setting and that it takes time to communicate smoothly, it would be required to introduce activities for building rapport among team members and checking the interim state of teamwork tasks.

### Limitations and Directions for Future Research

Regarding the study’s limitations and directions for future research, five aspects are observed. First, although this program surveyed the need for career education as perceived by career education experts for program development, it did not survey the need for a career adaptability program as perceived by college students. Future research should recruit participants considering their characteristics based on their career development status and develop a program reflecting participants’ needs. Second, the career adaptability program developed consisted of educational programs that correspond to the overall sub-factors of career adaptability for 1st-year students. This is because the program’s development process focused on an educational program best suited for the related university curricula within the limited university schedule. In the future, it would be necessary to provide a customized program composed of a common program and an optional program for learners to choose as necessary according to the result of the career adaptability diagnosis. Furthermore, there is a need for developing separate career adaptability programs for college students based on majors and education level. This is because the contents of a career program may vary depending on the major and the career development stage and tasks may vary depending on education level. Third, this study primarily used quantitative analysis to test its effectiveness. While a satisfaction survey was conducted and participants had time to share their experiences, the data collected were insufficient for qualitative analysis. Follow-up research should encourage participants to write an experience report after every session for continuous program updates so that the program contents and operation method can continuously be revised and supplemented. Fourth, program effectiveness was tested by examining the intergroup differences in pre-post-career adaptability scores. However, to monitor whether career adaptability improvement is maintained, it would be desirable to introduce follow-up tests in addition to pre-posttests. Fifth, an attempt was made in this study to develop and apply a real-time online career education program. However, it would be necessary to ensure an online learning management system that can overcome online-specific difficulties such as limited interaction between instructors and students in contact-free situations. Future research might as well develop an on/offline blended career adaptability program and prepare a platform for developing and distributing various online career education programs.

### Practical Implications

This study contributes to the literature on improvement of college students’ career adaptability. Based on the results of its effectiveness verification, the following practical application strategies are proposed.

First, an educational program linked with a career adaptability diagnosis tool was developed in this study to improve undergraduate students’ career adaptability. Developing an educational program linked with the sub-factors of the career adaptability scale for college student made a personalized intervention possible. In turn, this improved insufficient factors in each student’s career adaptability profile. Since this program included a wide range of career adaptability factors required to solve the career development tasks of college students, the student-centered program contents can be selectively applied in a manner that improves factors the student lacks as detected by the career adaptability diagnosis test.

Second, this program considered “knowledge and recognition of self and work environment,” “self-directed coping related to career behavior,” and “environmental interaction for career decisions and adaptation” as domains to improve career adaptability. These career adaptability domains apply to university students who have to determine a career path and accomplish certain tasks, and to job-seeking students in the school-to-work transition and to employees envisaging turnover. However, the results of effectiveness verification of this program are limited to college students. Follow-up studies need to examine the effectiveness of this program when applied to population groups other than college students.

## Conclusion

Overall, the findings indicated that a career adaptability educational program was effective in improving college students’ career adaptability. The program’s content areas and activities were organically linked with college students’ career development task area and career adaptability sub-factors. Moreover, students who participated in the career adaptability improvement program, developed in this study, scored higher in overall career adaptability than those who participated in the existing career education program. Various career adaptability sub-factors required for carrying out college students’ career development tasks could be applicable in career education and counseling settings because they include a range of career problems encountered by college students.

## Data Availability Statement

The raw data supporting the conclusions of this article will be made available by the authors, without undue reservation.

## Ethics Statement

The studies involving human participants were reviewed and approved by Dankook University. The patients/participants provided their written informed consent to participate in this study.

## Author Contributions

JK conceived, designed, and awarded the grant for the study, analyzed the data, and wrote and discussed the manuscript.

## Funding

This work was supported by the Ministry of Education of the Republic of Korea and the National Research Foundation of Korea (NRF-2020S1A5A8042232).

## Conflict of Interest

The author declares that the research was conducted in the absence of any commercial or financial relationships that could be construed as a potential conflict of interest.

## Publisher’s Note

All claims expressed in this article are solely those of the authors and do not necessarily represent those of their affiliated organizations, or those of the publisher, the editors and the reviewers. Any product that may be evaluated in this article, or claim that may be made by its manufacturer, is not guaranteed or endorsed by the publisher.
